# Gardens reduce seasonal hunger gaps for farmland pollinators

**DOI:** 10.1098/rspb.2024.1523

**Published:** 2024-10-23

**Authors:** T. P. Timberlake, N.E. Tew, J. Memmott

**Affiliations:** ^1^ School of Biological Sciences, University of Bristol, 24 Tyndall Avenue, Bristol BS8 1TQ, UK

**Keywords:** pollinator, garden, phenology, floral resource, urban, bumblebee

## Abstract

Gardens can benefit pollinators living in surrounding farmland landscapes, but the reason for their value is not clear. Gardens are no different from many semi-natural farmland habitats in terms of the *quantity* of floral resources (pollen and nectar) they produce, but the *timing* of their resource supply is very different, which may explain their value. We show that gardens provide 15% of overall annual nectar in farmland landscapes in Southwest UK, but between 50% and 95% during early spring and late summer when farmland supplies are low. Gardens can therefore reduce seasonal nectar gaps experienced by farmland bumblebees. Consistent with this pattern, bumblebee activity increased in gardens relative to farmland during early spring and late summer. An agent-based model reinforces this point, showing that *timing*, not *quantity*, of garden nectar supply enhances bumblebee colony growth and survival in farmland. We show that over 90% of farmland in Great Britain is within 1 km of a garden and therefore positive actions by gardeners could have widespread spillover benefits for pollinators across the country. Given the widespread distribution of gardens around the world, we highlight their important interplay with surrounding landscapes for pollinator ecology and conservation.

## 1. Introduction

Reversing global pollinator declines will require new approaches to the management of anthropogenic landscapes and a better understanding of how pollinators use them. Farmland is by far the largest anthropogenic landcover, encompassing around half of the world’s habitable land area and more than 70% in some countries such as the UK [1]. In contrast, urban areas (defined in their broadest sense) cover only around 2% of the Earth’s land surface [2] and 8% of the UK [3], but are scattered widely and relatively uniformly across most countries according to consistent and largely predictable laws of residential scaling and human dispersion [4,5] (electronic supplementary material, figure S1). Urban areas are therefore much more widely distributed than their land area would suggest, making it important to consider their impacts—both positive and negative—on surrounding biodiversity, especially for mobile organisms such as pollinators.

Floral resources (pollen and nectar) are the primary food source for most pollinators and thus can be a major factor limiting their populations [6,7]. Decades of agricultural intensification have led to a decline in the quantity, diversity and temporal continuity of floral resources [8,9], likely contributing to parallel rates of decline in many pollinator taxa [10,11]. Reversing pollinator declines requires us to understand where floral resources are located—both in time and space—and how pollinators are using them, so that we can manage landscapes to increase the quantity, accessibility and seasonal continuity of food for pollinators [12]. Floral resources have been measured independently for both farmland [13–16] and urban landscapes [17–19]. However, a realistic landscape-level assessment of floral resources needs to consider the contributions of both farmland and urban areas together, as flying insects can move freely between them, using resources from both.

Urban areas are often considered hotspots of pollinator diversity, supporting a higher abundance and richness of some taxa than surrounding farmland ([20–24], but see [25]). Most of this urban pollinator diversity is concentrated in the residential gardens around domestic properties (known as ‘yards’ in the USA), which provide up to 85% of urban floral resources in UK cities [18]. The benefits of gardens to pollinators can extend into surrounding farmland, with higher population densities of farmland pollinators recorded in proximity to gardens [26–28]. Despite the well-established benefits of gardens for pollinators, the mechanisms underlying this effect are not well understood. The high density of suitable nesting habitats within gardens provides one possible explanation [29], but the floral (food) resources provided by gardens could be even more important, as food is frequently limiting for pollinators [6,30,31]. This is consistent with evidence showing bumblebee colonies growing faster, producing more sexuals and surviving longer (all associated with better food supply) in proximity to urban areas [32]. However, the annual nectar production of gardens [18] is actually similar to that of semi-natural farmland habitats such as hedgerows and field margins [13]. Moreover, their low coverage of farmland landscapes means their total contribution to landscape nectar supply is relatively small. Therefore, we hypothesize that it is not the *quantity* of floral resources provided by gardens that makes them so important to pollinators, but instead the *timing* of their resource supply.

The supply of floral resources in temperate farmland varies greatly through the year, with alternating periods of resource surplus and deficit [13,14,16,33]. These periods of deficit, or ‘hunger gaps’, are known to have a deleterious effect on pollinator populations, particularly in early spring and late summer [28,34–37]. In contrast to farmland, the supply of floral resources in clusters of gardens is more diverse, stable and long-lived, with a smoother overall phenological profile [19]. Thus, while gardens may provide a relatively small proportion of annual pollen and nectar supply in rural landscapes, they could be disproportionately important in providing these resources at times when it is otherwise scarce, reducing crucial resource gaps.

To test this hypothesis experimentally, it would be necessary to manipulate garden floral resource supply in replicate large-scale study landscapes—an undertaking with considerable logistical challenges. An alternative approach is to predict the patterns of floral resource availability, pollinator behaviour and pollinator population dynamics that we would *expect* to see if the hypothesis were true, and test whether these patterns hold. In the case of our hypothesis—that gardens buffer farmland pollinators against seasonal shortages in floral resources—we would expect to see the following patterns: (i) gardens reduce temporal gaps in the floral resource supply of farmland landscapes; (ii) pollinators increase their use of garden floral resources during periods of low farmland resource availability; (iii) pollinator populations respond more strongly to changes in the *timing* than the total *quantity* of garden resources. Using bumblebees as a model group, and Southwest UK as a study region, we test these three hypotheses with a combination of empirical field data and agent-based modelling. To investigate how widespread these benefits of gardens could be, we also ask: (iv) how much of Great Britain is within reach of a garden for a foraging pollinator?

## 2. Methods

### (a) Question 1: do gardens reduce seasonal gaps in the resource supply of farmland landscapes?

To determine whether floral resources provided by gardens have the potential to reduce seasonal gaps in the food supply of farmland pollinators, we quantified the nectar production of three farmland landscapes in Southwest UK (electronic supplementary material, figure S2) throughout an entire flowering season and estimated the additional nectar provided by small clusters of rural gardens within these landscapes. Although nectar provides only one component of pollinator nutrition (the carbohydrates required for energetic processes) and various lines of evidence show that pollen (rich in protein and lipids) is an equally important determinant of the nutrition and foraging behaviour in bees [38–40], we focus on nectar because it is the only floral resource that has been quantified in a wide range of both farmland and garden plants [8,18]. Nevertheless, we expect to see similar seasonal patterns for both pollen and nectar because these two resources are closely correlated at both the floral unit scale (owing to their covariance with flower size) and the landscape scale, as the supply of both resources is largely driven by floral abundance [41].

#### (i) Recording nectar phenology in farmland

Farmland floral abundance was recorded during 2017 in three medium-sized (142–213 ha) mixed farms in Southwest UK (electronic supplementary material, figure S2). None of the farms was under any form of pollinator-friendly management and all three farms were more than 5 km from a major urban area (i.e. town or city), but within foraging range of rural villages. The sites contained a mixture of pasture and arable land, with fields separated by hedgerows, field margins or semi-natural woodland. As is typical of much of the UK, a small number of human settlements and associated gardens as well as artificial surfaces (roads, buildings, etc.) were also found within each of the farmland study landscapes (electronic supplementary material, table S1). The seasonal nectar production and habitat composition of these three study sites aligns with the range of variation seen in a wider study of 12 farmland landscapes across Southwest UK ([28]; electronic supplementary material, figure S3). Thus, they can be considered generally representative of the region, and of European farmland more broadly, where similar patterns of seasonal nectar production are found ([14–16]; electronic supplementary material, figure S4).

From March to October 2017, each of the three farms was visited once per week to record floral abundance in each type of semi-natural habitat (permanent pasture, semi-natural woodland, hedgerows and field margins). These habitats are clearly delineated in most farms in this region and exhibit consistent differences in their quantity and timing of resource availability [28], so represent practical and informative floral resource sampling units. On each sampling occasion, six 50 m transects were randomly placed in each semi-natural habitat type (e.g. 24 transects in total, for a farm with four habitat types) and 10 quadrats of 1 m^2^ were distributed along the transect at 5 m intervals. The number of open floral units of each flowering plant species within or directly above each quadrat was recorded to provide an estimate of the number of open floral units present in each square metre of habitat at a given point in the year. For trees and shrubs, all floral units in a 5 m vertical column above the quadrat were counted. Above this, the tree’s height within the vertical column was estimated with a clinometer and the floral abundance values were multiplied up accordingly [8]. A floral unit was defined as one or multiple flowers that can be visited by insects without flying [42]. The floral unit values were then multiplied by the mean floral sugar production of each species to obtain an estimate of the grams of sugar per unit area per 24 h period for each habitat. Values for the nectar sugar production of farmland species were from Baude *et al*. [8] and Timberlake *et al*. [13], who recorded the sugar production of 125 flowering plant species that collectively made up >98% of all floral units on the farms. In brief, nectar was collected by enclosing at least 10 individual flowers (generally from two different sites on different days) in netting bags for 24 h to exclude insects, and then extracting their nectar using micro-capillary tubes (Minicaps, Hirshmann, Eberstadt, Germany). The sugar concentration (grams of sucrose per 100 ml solution) of each sample was measured using a hand-held refractometer (Eclipse, Bellingham and Stanley, Tunbridge Wells, UK). Although nectar secretion by individual flowers is a plastic trait that varies with both biotic and abiotic conditions [43], this flower-level variation has a limited impact on landscape-level nectar availability that is predominantly determined by the number of flowers in the landscape [41].

#### (ii) Recording nectar phenology in gardens

Garden floral abundance was recorded during 2019 in 59 urban gardens in the city of Bristol, South West UK. Although gardens were sampled in a different year from farmland, the mean annual temperature, rainfall and growing season of these 2 years were similar (electronic supplementary material, figure S5) and previous data from this region show a similar pattern of nectar phenology between years [13]. Residential gardens included the land adjacent to and associated with each domestic property (both front and back gardens), irrespective of whether it was paved, vegetated or used as a driveway. In total, gardens ranged in size from 31.3 to 407.7 m^2^ (mean 156.4 m^2^ ± 12.7 s.e.) and their selection was stratified by both geographical location and neighbourhood income (see [19] for further details). From March to October 2019, each garden was visited once per calendar month to record floral abundance by counting the number of open floral units of each flowering plant species within or directly above each garden. In contrast to farmland habitats, sampling along a 50 m transect is not practical in residential gardens, which exist as small discrete patches of a single land use type. Instead, floral units were either counted individually using a handheld tally counter or estimated by sub-sampling (e.g. for flowering shrubs and trees and flower-rich lawns). The floral unit values were then multiplied by the mean floral sugar production of each species to obtain an estimate of the grams of sugar per unit area per 24 h period for each garden. Values for the nectar sugar production of garden species were from Baude *et al*. [8], Hicks *et al*. [17] and Tew *et al*. [18,19], who quantified the sugar production of all 636 flowering plant species present in these gardens using the methods described in the previous section. Variation in garden nectar supply stabilizes at the point of around 20 gardens [19], suggesting that 59 gardens is a sufficient sample from which to characterize the average nectar profile for gardens in Bristol. Furthermore, values of average garden nectar production show no significant differences between UK cities [18], suggesting that the patterns recorded in our study are likely to be broadly representative of gardens at a national scale.

#### (iii) Merging the farmland and garden datasets

The floral abundance surveys in garden and farmland habitats generated data in the following units: grams of nectar sugar m^−2^ day^−1^. Regular repeated measurements of this common currency throughout the flowering season allowed us to characterize the phenology of nectar supply in high resolution for each landcover and to compare between them. A generalized additive model (GAM) was used to model this smooth, nonlinear trend in nectar availability over time. A thin-plate regression spline was used to model day of the year, with the degree of smoothing selected using the default generalized cross-validation method [44]. The outputs from these models (grams of nectar sugar m^−2^ day^−1^) were multiplied by the area of each habitat in a 1 km^2^ area of farmland landscape (including gardens that comprised 1.9% ± 0.01 s.e. of our study landscapes) to compare their contributions to landscape-level nectar supply. Floral abundance was not directly measured in rural gardens owing to a lack of nectar data at the time of farmland sampling in 2017. We therefore make the assumption that the seasonal pattern of nectar supply is the same in rural village gardens as it is in gardens within the nearby city of Bristol. Previous studies show high consistency in plant species richness, diversity and composition [45] as well as in total nectar supply [18] in gardens across different geographic and climatic zones of the UK, suggesting our assumption is likely to hold true.

#### (iv) Identifying resource gaps

Periods of nectar deficit (‘hunger gaps’) for farmland bumblebees have been previously recorded in farmland landscapes in Southwest UK [13] and we wanted to investigate the potential for garden nectar supplies to reduce these gaps. For each week of the flowering season, the total nectar sugar production of the farmland landscape at a 1 km^2^ scale (taken from the GAM outputs—see above) was divided by the estimated number of individuals of the three most common bumblebees in UK farmland (*Bombus terrestris, B. pascuorum* and *B. lapidarius*). Information on the typical colony size and density (per km^2^ of farmland) was taken from Dicks *et al*. [46], while the phenology of each bumblebee species was taken from BeeWalk transect data in 2017 [47]. BeeWalk is a standardized monitoring scheme hat collects data on the abundance and distribution of bumblebees in Britain. Fixed transects with clearly defined habitat composition are systematically surveyed by trained volunteer recorders once per month from March to October. This provided an estimate of the grams of daily sugar available to each individual bumblebee throughout the year. Overlain on the same graph, we added the estimated sugar requirements of an individual bumblebee (accounting for foraging costs and the elevated demands of queens in the early spring) using data from Heinrich [48] and Rotheray *et al*. [49]. This approach enabled us to identify periods of the year when there is likely to be a nectar surplus or deficit in farmland landscapes, and compare the extent of nectar deficits with and without the contribution of garden nectar supply.

### (b) Question 2: do pollinators increase their use of gardens during periods of low farmland resource availability?

The purpose of this analysis was to establish whether there is a seasonal pattern of bumblebee activity in garden versus farmland habitats and to assess whether bumblebee activity in gardens increases during periods of the year when garden resources are relatively more abundant than farmland. To establish seasonal patterns of bumblebee activity in farmland and gardens, we analysed occurrence records of four common farmland bumblebee species (*B. terrestris*, *B. pascuorum*, *B. lapidarius* and *B. pratorum*) from BeeWalk transect data collected between 2008 and 2019 ([47]; see §2a(iv)). We included only records from the South West region of the UK (counties of: Bristol, Cornwall, Dorset, Devon, Gloucestershire, Somerset and Wiltshire) to minimize any confounding effects of latitudinal differences in phenology. Records were filtered to include transect sections whose primary habitat designation was strictly related to either farmland or garden. Any transect sections classified as primarily agricultural but secondarily garden (99 records in total) were removed to avoid ambiguity; this left us with a total of 493 km of purely farmland transect sections and 168 km of garden-associated transect sections. For each of the two habitat types (i.e. farmland and garden), we calculated the total number of worker and queen bumblebees recorded in each month of the year for each of the four bumblebee species (hereafter referred to as bumblebee activity). Male bumblebees were excluded from the analysis because their foraging patterns and phenology of activity are different from those of females. Total observations for each species were divided by the total length of transect recorded in each habitat type so that results were scaled by sampling effort. Activity values were therefore expressed as the mean number of bees recorded per kilometre of transect. For each month of the year, we divided bumblebee activity values in gardens by activity values in farmland (i.e. the ratio of garden : farmland bumblebee activity) to identify which periods of the year bumblebees were relatively more active in gardens, and *vice versa*.

### (c) Question 3: do bumblebees respond more strongly to changes in the timing than the total quantity of garden resources?

To test the influence of garden nectar supply on farmland bumblebee population dynamics and disentangle the effects of resource quantity (how *much* nectar) from the effects of resource phenology (*when* the nectar is available) we ran an *in silico* experiment using the agent-based model ‘BEE-STEWARD’ developed by Twiston‐Davies *et al*. [50] implemented in *Netlogo* [51]. The model simulates the foraging behaviour, life history and colony growth of bumblebees foraging for nectar and pollen in a temporally dynamic, spatially explicit, user-defined landscape. Bee foraging activity is modelled based on the detection probability and attractiveness of individual forage patches (influenced by their size and distance from nest sites), flower handling time (affected by corolla depth and resource depletion) and sugar concentration (this can be defined by the user). Colony growth is an emergent property of floral resource acquisition (as well as other environmental factors) and is based on empirical data (see electronic supplementary material, figure S6 for an overview of model structure). BEE-STEWARD, and related models, have been widely used in pollination ecology research as they enable users to alter the spatial, temporal and nutritional features of floral resources (as well as other aspects of the environment) and investigate the resulting impacts on bee colony dynamics (see https://beehave-model.net/publications/).

Our model landscapes were based on 12 real circular farmland landscapes with a 1 km radius (3.14 km^2^ area) in Southwest UK that were characterized and mapped by Timberlake *et al*. [28] (electronic supplementary material, figures S2 and S7). Each of these study landscapes had at least some garden present, ranging from 0.2% to 5.9% coverage, with a mean of 1.9% ± 0.01 s.e. (electronic supplementary material, table S1). The quantity and phenology of nectar supply in each habitat were based on empirical data from our study (see §2 above).

In each of the 12 model landscapes, we ran four alternative simulations to test the importance of gardens. These were: (i) gardens present as normal; (ii) gardens removed completely; (iii) gardens replaced with pasture (the most common land use in this region, which could also be considered an analogue of a simple lawn garden); and (iv) gardens replaced with an alternative landcover of identical total floral resource value but with the phenological pattern of pasture (i.e. the same total resources but distributed differently through the year), to test the role of phenology *per se*. All other features of the landscape, including pollen availability and nesting site availability, were kept identical amongst treatments. In real landscapes, there will inevitably be other population-limiting factors such as pollen availability, nesting sites, predation and weather. However, we focus only on the impact of nectar availability in otherwise benign conditions. Experiments were run with *Bombus terrestris* only and began with 500 queens, as is the default for BEE-STEWARD. Mean emergence date and standard deviation were kept consistent between all sites, using default values from BEE-STEWARD (01 April ± 28 days s.d.).

We selected five different population response variables to provide us with a broad view of the mechanisms driving population change. These were: (i) colony density throughout the flight season (recorded on the 15th day of each month); (ii) maximum number of colonies produced per year; (iii) total number of bees produced per year; (iv) number of new queens produced per year; and (v) percentage spring queen survival. The model was run 20 times for each treatment–farm combination, and for each simulation run we calculated the mean value for each response variable over the first 5 years of the simulation. The great majority of populations persisted longer than 5 years, so 5 years was taken as a cut-off for the period of relative population stability. For each of the different measured endpoints/response variables, a generalized linear mixed model (GLMM) with the appropriate error distribution was used to test for differences between the treatments, with ‘treatment’ as a fixed factor and ‘farm’ as a random factor.

### (d) Question 4: how much of Great Britain is within reach of a garden for a foraging pollinator?

To investigate the spatial arrangement and ubiquity of gardens across Great Britain (England, Scotland and Wales), we mapped the distribution of urban areas with GIS tools (ArcMap 10.6.1) using land cover data from the Centre for Ecology and Hydrology (2015). This landcover category includes both dense urban areas (e.g. towns and cities) and suburban areas with mixed urban and vegetation signatures (e.g. small villages and associated gardens). Buffers were drawn around urban land at radii of 1000, 750, 500 and 250 m. These distances were chosen to reflect the approximate foraging distances of bumblebees and honeybees (750–1000 m), relatively large-bodied solitary bees (500 m) and smaller-bodied bees (250 m) [52]. A buffer of 1000 m contains all land that is within 1000 m of an urban area. There is no high-resolution map of gardens at a national scale, but given that urban areas contain a high density of regularly spaced gardens (29.5% of all urban land in Great Britain is garden; ONS 2019), we assume that a buffer around urban land is roughly equivalent to a buffer around gardens. The area of land within urban buffers was calculated in ArcMap and subdivided into the three countries comprising Great Britain (England, Scotland, Wales) and the nine geographic regions comprising England (electronic supplementary material, figure S8).

### (e) Data analysis

All statistical analyses were performed in R v. 4.3.1 [53]. Generalized additive models (GAM) were performed in the package *mgcv* [44] and GLMMs in the package *nlme* [54]. Pairwise comparisons between treatments in GLMM were calculated and visualized using the package *marginal effects* [55].

## 3. Results

### (a) Question 1: do gardens reduce seasonal gaps in the resource supply of farmland landscapes?

**Figure 1.**
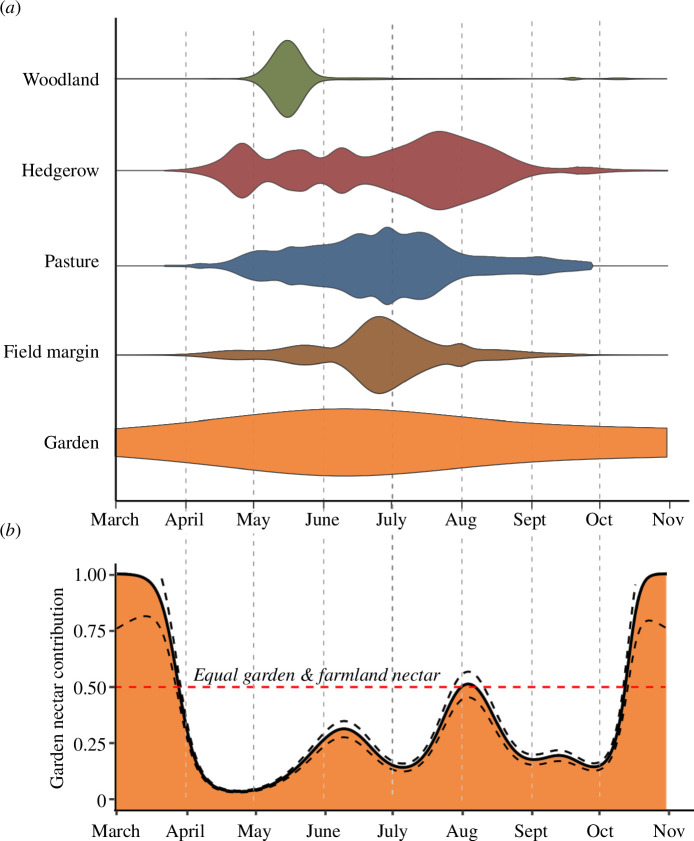
Nectar phenology profiles of woodland, hedgerows, pasture, field margins and gardens (*a*), based upon the daily sugar production of each habitat. Values from the three farms are combined to model a smooth overall trend in nectar production through the year. Results are normalized for each habitat so do not convey information on overall nectar production, only the phenological pattern of resources; for absolute values, see electronic supplementary material, figure S9. (*b*) The proportional contribution (±s.e.—dashed lines) of gardens to total landscape nectar supply on the three study farms. The horizontal dashed line shows the point at which gardens are providing exactly 50% of total landscape nectar supply. Areas above this line (in March and October) are periods when gardens supply the majority of landscape nectar. Absolute values of garden and farmland nectar supply are shown in electronic supplementary material, figure S10. Although the timing of peaks and troughs will differ amongst regions, farmland habitats are likely to always display a more variable phenology than gardens owing to their low species diversity and homogenous management practices.

Gardens provided just 15% (±1.8% s.e.) of the annual nectar supply of our farmland landscapes, whereas the remaining 85% (±36% s.e.) was provided by hedgerows, woodland, pasture and field margins. However, while these semi-natural habitats contributed the majority of farmland nectar, their supply was relatively short-lived and highly variable through the year, being provided by just a handful of species (electronic supplementary material, table S2). In contrast, gardens provided a small but consistent and diverse supply of nectar throughout most of the year, buffering some of the variability of semi-natural habitats (figure 1*a*).

**Figure 2.**
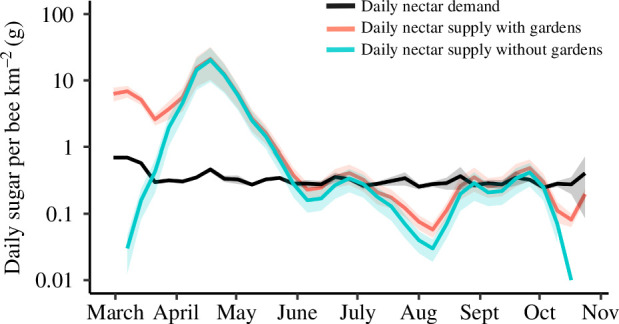
Nectar deficits for farmland bumblebees are substantially reduced when the contribution of nectar from rural gardens is taken into account. The *y*-axis shows the total landscape nectar supply (in a 1 km^2^ area) with and without the contribution of nectar from gardens (pink and blue lines, respectively), divided by the estimated number of bumblebees present in a 1 km^2^ area. Bumblebee abundance is based on typical values of colony density and colony size of the three most common farmland bumblebees from [46]. Values are the mean of the three study farms ± s.e. (shaded ribbons). Overlain on the graph is the estimated mean daily sugar requirement (black line ± s.e.) of a *B. terrestris* individual at each point in the year, as taken from Rotheray *et al*. [49]. Points where the coloured lines dip below the black line are periods of nectar deficit for bumblebees. Note the *y*‐axis is plotted on a log_10_ scale.

The proportional contribution of gardens to total nectar supply in the three study landscapes was greatest in the early spring and late summer, when farmland nectar supplies were low (figure 1*b*). Gardens supplied a total of 72% (±11% s.e.) of all nectar during March, 51% (±6% s.e.) in early August and 93% (±18% s.e.) in the second half of October. When the nectar contribution of gardens (which cover 1.9% of farmland landscapes) was included in the total landscape-scale nectar supply, the early spring nectar deficit for bumblebees disappeared and the late-summer nectar deficit was substantially reduced (figure 2).

### (b) Question 2: do pollinators increase their use of gardens during periods of low farmland resource availability?

**Figure 3.**
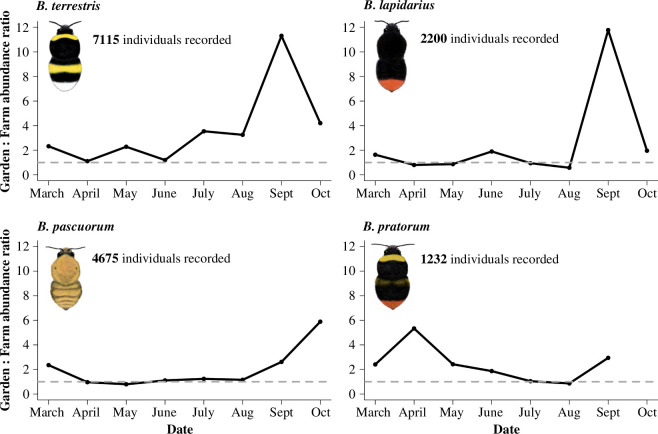
The ratio of garden : farmland bumblebee activity (measured as their relative abundance on transect recordings) shows a strong seasonal pattern, with most species showing a higher relative activity in gardens during early spring and late summer, than in the mid-summer. Data are taken from BeeWalk transect recordings in South-West UK [47] and are scaled by kilometre of transect in each habitat, to account for differences in sampling effort. Results show the activity of workers and queens but not males, as their foraging needs differ. Dashed grey lines mark the points of equal activity between gardens and farmland; any values above these lines represent a higher activity in gardens than farmland. Image credits: Bumblebee Conservation Trust.

Overall, bumblebees were relatively more active (i.e. frequently encountered) in gardens than in farmland habitats, with between 1.2 and 2.8 times more bees recorded per kilometre of garden transect, compared with farmland (electronic supplementary material, table S3). The relative activity of bumblebees in the two habitats did not remain constant throughout the year, however; activity was highest in gardens during the early spring and late summer (figure 3). Bumblebees were at least twice as active in garden than in farmland habitats during the early spring (March/April) but the activity of most species approached parity in the two habitats during the mid-summer. In the late summer (August–October), bumblebee activity rose to between 4 and 12 times higher in gardens than in farmland (figure 3), reflecting a similar seasonal pattern to garden nectar contributions (figure 1*b*). Although these correlative patterns do not imply causation, they are consistent with our hypothesis that bumblebees increase their use of garden floral resources during periods of low resource availability in farmland.

### (c) Question 3: do bumblebees respond more strongly to changes in the timing than the total quantity of garden resources?

**Figure 4.**
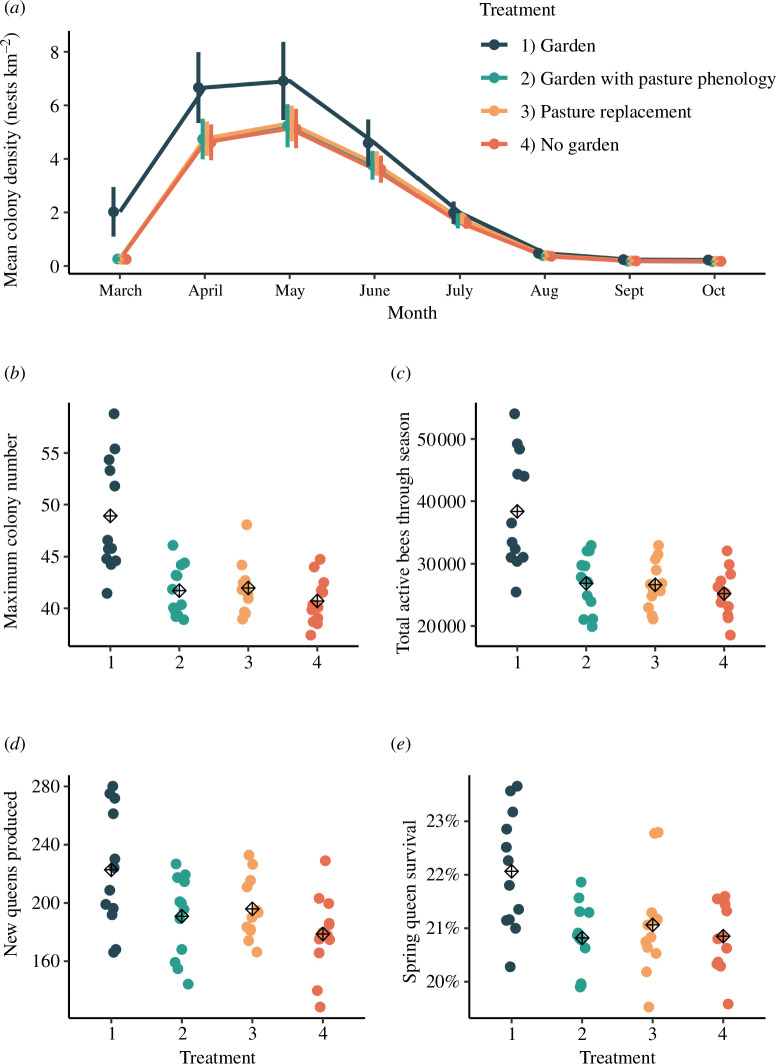
Results of the BEE-STEWARD model, which simulates bumblebee population dynamics in 12 study landscapes based upon empirical nectar phenology data. Population metrics recorded in the model included: (*a*) colony density through the year (mean nests per km^2^ ± s.d.); (*b*) the maximum number of bumblebee colonies recorded during the year; *c*) total number of active bees produced during the year; (*d*) number of new queens produced in the spring; (*e*) percentage of new queens that survive to establish colonies. The circles in panels (*b*–*e)* show the results for each of the 12 study landscapes (mean of 20 independent model runs) whilst the cross hairs show the mean of all 12 landscapes. Treatments include: 1) gardens present as normal (*Garden*); 2) gardens replaced with a landcover of identical annual nectar value but the phenological pattern of pasture, i.e. resources concentrated in the mid-summer (G*arden with pasture phenology*); 3) gardens replaced with pasture (*Pasture replacement*); and 4) gardens removed completely (*No garden*). In all cases, the ’gardens as normal’ treatment shows significantly higher values than all other treatments (electronic supplementary material, figure S11).

Despite gardens making up only 1.9% ± 0.01 s.e. of our study landscapes, BEE-STEWARD model simulations showed a 16.8% ± 6.7 s.d. decline in annual *B. terrestris* colony numbers when gardens were removed from the landscape (*t*_1,33_= −10.6, *p* < 0.0001) and a 14.2% ± 6.3 s.d. loss when replaced by pasture (*t*_1,33_= −9.0, *p* < 0.0001). Even when replaced with a fictional habitat of identical total nectar provisioning to gardens but a phenology profile matching pasture, annual colony numbers were still 14.7% ± 6.3 s.d. lower (*t*_1,33_= −9.3, *p* < 0.0001), demonstrating the importance of phenology in driving these patterns (figure 4; electronic supplementary material, figure S11). Percentage decreases were higher in the early spring, with up to 87% ± 8% s.e. declines in colony density (figure 4*a*). These patterns were driven by lower floral resource availability in the early spring and late summer, resulting in 5.6% ± 2.1 s.d. lower queen survival rates in the spring (*t*_1,33_= −3.5, *p* = 0.0004; figure 4*e*) and 19.7% ± 6.4 s.d. fewer new queens produced in the late summer (*t*_1,33_= −5.8, *p* < 0.0001; figure 4*d*). The total number of individual bees in each landscape was at least 30% ± 12 s.d. lower each year when gardens were absent or replaced, with implications for ecosystem service provisioning (*t*_1,33_= −12.2, *p* < 0.0001; figure 4*c*). These results are consistent with empirical data from the same 12 farms showing a significant correlation between *B. terrestris* colony density and the extent of garden cover [28].

### (d) Question 4: how much of Great Britain is within reach of a garden for a foraging pollinator?

**Figure 5.**
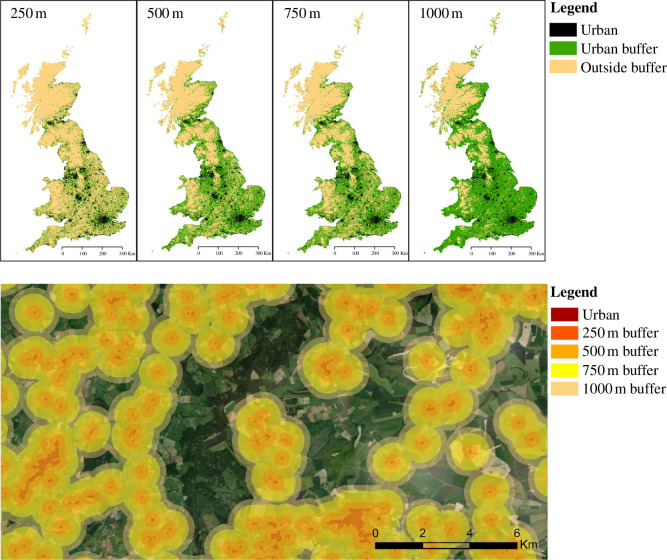
The regular pattern of urban areas across Great Britain means that pollinators are seldom far from a garden. The top panel shows urban landcover in Great Britain (black shading), with urban buffers (green shading) representing the extent of the country that is potentially within reach of these urban areas for pollinators of various different foraging ranges (see buffer distances at the top of each panel). The bottom panel provides an example of the distribution of human settlements in an area of Southwest UK (Imagery: Esri, DigitalGlobe). This characteristic pattern of scattered settlements means that although gardens cover only a very small land area, they are widely accessible to mobile organisms. Buffer distances are chosen to reflect the approximate foraging distances of bumblebees and honeybees (750–1000 m), relatively large-bodied solitary bees (500 m) and smaller-bodied bees (250 m) [52].

Urban areas cover only 8% of the UK but their spatial arrangement means that a substantial proportion of the country is within close proximity of them (figure 5; electronic supplementary material, table S4). By drawing buffers around each piece of urban land, we show that 68% of Great Britain (87% of England, 68% of Wales and 35% of Scotland) is within 1 km of an urban area and therefore potentially accessible to mobile pollinators such as bumblebees and honeybees that are able to forage over a 1 km radius [52]. Even for smaller-bodied pollinators with lower dispersal abilities such as solitary bees, much of the country is still within reach of an urban area; 48% of Great Britain and 64% of England are within 500 m of an urban area. For pollinators living in farmland landscapes, accessibility to urban areas is even higher: almost all farmland in Great Britain (91%) is within 1 km of an urban area. Only 7% of arable land and 11% of pasture is more than 1 km from an urban area.

## 4. Discussion

Our results show that that gardens provide a temporally stable supply of floral resources that has the potential to buffer the variability in farmland resource supplies and reduce seasonal nectar deficits for farmland bumblebees in Southwest UK. Empirical data show a pattern of bumblebee activity that is consistent with this effect, while an agent-based population dynamic model reveals the benefit of temporally consistent garden nectar resources for bumblebee colony growth and survival. Given the consistency among these different strands of empirical and modelled data and the ubiquitous nature of scattered gardens across Great Britain, we suggest that gardens have the potential to play a widespread role in buffering pollinators against seasonal gaps in the supply of farmland floral resources.

Our results indicate that the well-established positive effect of gardens on farmland pollinators [26–28,32] derives not so much from the *quantity* of their floral resource production, but from its *timing*. Gardens complement the more abundant but seasonally variable supply of farmland floral resources with their relatively low, but stable, supply at a landscape scale. Although the exact seasonal pattern of floral resource supply will differ among landscapes across the UK and elsewhere, temporal gaps are likely to be common in all modern simplified agricultural landscapes, as large blocks of homogenous habitat with low floral diversity mean a handful of species tend to dominate flowering for short periods. Highly variable supplies of floral resources through the year, characterized by seasonal gaps, have been recorded in farmland settings as diverse as Poland [14], Switzerland [15], the Netherlands [16] and North America [9,56]. By contrast, the floral diversity of gardens is high [45,57], with 56 plant species responsible for 80% of the nectar supply in our study gardens ([19]; electronic supplementary material, table S2) compared with only eight species in farmland in Southwest UK [13] and 12 species in the UK as a whole [8]. In contrast to farmland, many of the species providing nectar in gardens are exotics, some of which may be inaccessible or unfavourable to certain pollinators [58]. However, 96% of the top-50 nectar-provisioning plants in our study gardens are listed as ‘bee friendly’ by the UK Bumblebee Conservation Trust (electronic supplementary material, table S2). Moreover, the high diversity of nectar sources in gardens means the presence of a few unfavourable nectar sources is less of a problem than it would be in farmland. Although there is a high degree of variation amongst individual gardens in their floral resource supplies, gardens vary in a relatively independent fashion owing to different management decisions and turnover in plant communities [19]. The result is that clusters of multiple gardens collectively average out floral resources, through a portfolio effect, to provide pollinators with a more stable and continuous supply of resources than is otherwise available in farmland habitats [19]. This may help to explain the relatively lower rates of bee decline in landscapes associated with urban expansion over the past 80 years in England [59].

Data from systematic BeeWalk transects show a pattern of bumblebee activity that is consistent with our hypothesis—that bumblebees increase their foraging activity in gardens during periods when farmland floral resources are low. This reflects findings from the UK [41,60], Germany [61] and the USA [62], where pollinators shift their foraging diet towards exotic species in late summer and autumn, when resources are more limiting. These additional late-season floral resources can extend the period of pollinator activity, with implications for both pollinator populations and plant reproduction [63]. Although gardens typically cover a very small proportion of the landscape in comparison with farmland, bumblebees are able to locate and utilize the flower-rich patches within them, as demonstrated by the presence of garden plant pollen on 50% of bumblebees captured in farmland sites, increasing to 70% in the late summer when farmland nectar is most limited [41]. As bumblebees are adept at locating and exploiting small flower-rich patches [64,65], we would expect them to utilize gardens as a source of floral resources in much the same way as they would hedgerows or areas of remnant woodland in bloom. By offering regularly spaced and temporally consistent hubs of floral resources, gardens may provide an important and largely unrecognized ecosystem service to surrounding agriculture by sustaining and enhancing pollinator populations in otherwise impoverished farmland landscapes [66]. Indeed, the results from our modelling exercise show that small areas of garden within a farmland landscape can support 30% more bumblebee workers than farmland alone, potentially translating into an increased provision of pollination service. The same benefits may not apply to pollinators with more specialist diets, however, as they are less likely to exploit floral resources from exotic garden plants.

Results from the bumblebee population dynamic model BEE-STEWARD demonstrate the value of this temporal complementarity between farmland and garden resource supply for bumblebee colony development and survival. The additional resources provided by gardens in the early spring and late summer coincide with two particularly important stages of bumblebee colony development, namely the emergence of queen bumblebees and the establishment of new colonies in the early spring, and the production of new queens in the late summer—which need to build up energy-rich reserves in advance of hibernation [67,68]. Consequently, the removal of seasonally consistent garden nectar resources from modelled farmland landscapes reduced the survival of early-emerging queens and the number of new queens produced in the late summer. Owing to a lack of data on the pollen production of garden plants, we were unable to manipulate pollen resources in the model in the way that we did for nectar, and are therefore likely to be underestimating the true value of gardens for bumblebee colonies. Pollen plays a very different nutritional role from that of nectar and it is required by pollinators in different quantities at different times of year [38] making it difficult to conclude exactly how the additional pollen provided by gardens would benefit pollinators. Nevertheless, the supply of farmland pollen shows a very similar seasonal pattern to that of nectar—with both being largely determined by the enormous seasonal variation in floral abundance [41]—so we expect gardens to smooth the seasonal variation in pollen supply in much the same way as they do for nectar.

Approximately half of the Earth’s habitable land is farmland, and much of this is interspersed with low-density scattered human settlements [5]. This suggests that the widespread proximity to urban areas that we report in this study may be a common feature across much of the world. Indeed, snapshots of four diverse agriculturally dominated regions (Northern France, North-East United States, Central Nigeria and Eastern China) all show a remarkably similar patterns of farmland interspersed with scattered human settlements (electronic supplementary material, figure S1). Although gardening practices vary among cultures, there is a growing body of literature to show that gardens can be valuable pollinator habitats in different countries, especially in Europe and North America [22,57,61,69]. Furthermore, a widespread desire of gardeners to intentionally select plants that give a long and continuous flowering display through the year is aided by the availability of exotic species and horticultural cultivars that are likely to extend the flowering season [70,71]. Even in regions of the world where gardens are primarily used for growing food, the high diversity of flowering crops still provides a valuable source of food for pollinators [72]. However, the patterns reported in our study will not apply in regions where urban areas are devoid of domestic gardens, or where few flowering plants are incorporated into them.

In conclusion, our study underscores the value that gardens can provide for pollinators—particularly bees—and demonstrates an important mechanism by which this can occur: —through increasing the phenological continuity of farmland floral resources. We emphasize the widespread positive influence that citizens might have on pollinators by managing their gardens to ensure a stable, long-lived supply of floral resources. Gardens that provide numerous flowers in the late summer (through targeted planting or mowing regimes) are likely to have the greatest benefit for bumblebees, as this is the period of greatest nectar deficit in UK farmland. The spatial arrangement of gardens across a landscape also plays an important role in their value to pollinators, with scattered rural gardens being the most likely to benefit farmland pollinators. We argue that a landscape-level approach to pollinator conservation needs to consider farmland and urban areas together, taking into account the interactions between them and the threats and benefits they collectively pose to pollinators.

## Data Availability

Data available via the Dryad digital repository [73]. R code available from Zenodo [74]. Supplementary material is available online [75].
